# Characterization and Antimicrobial Activity of PLA-Laminated PBAT/TPS Films Incorporated with Silver Nanocomposites

**DOI:** 10.3390/foods15122132

**Published:** 2026-06-13

**Authors:** Khwanchat Promhuad, Muenfun Papoompruk, Phatthranit Klinmalai, Nathdanai Harnkarnsujarit

**Affiliations:** 1 Department of Packaging and Materials Technology, Faculty of Agro-Industry, Kasetsart University, 50 Ngam Wong Wan Rd., Latyao, Chatuchak, Bangkok 10900, Thailand; khwanchatpromhuad@gmail.com (K.P.); papoompruk.m@gmail.com (M.P.); 2 Faculty of Agro-Industry, Chiang Mai University, Samut Sakhon 74000, Thailand; 3 Center for Advanced Studies for Agriculture and Food (CASAF), Kasetsart University Institute for Advanced Studies (KUIAS), Kasetsart University, 50 Ngam Wong Wan Rd., Latyao, Chatuchak, Bangkok 10900, Thailand

**Keywords:** polylactic acid (PLA), modified starch, silver nanoparticles, active packaging, antimicrobial activity

## Abstract

Multilayer packaging—engineered by integrating complementary materials such as plastics, paper, and aluminum—has become a cornerstone technology for enhancing shelf life, minimizing spoilage, and reinforcing the mechanical integrity of packaging formats including films, pouches, and bottles. In this study, a laminate was developed by thermally bonding polylactic acid (PLA) with a poly(butylene adipate-co-terephthalate) (PBAT)/thermoplastic starch (TPS) matrix embedded with silver nanoparticles (Ag-NPs) at 0–3 wt.%. The resulting structures were systematically evaluated for their barrier performance, physicochemical characteristics, and antimicrobial functionality. Fourier-transform infrared (FTIR) spectroscopy confirmed the absence of chemical interactions between Ag-NPs and the polymer matrix, indicating physical dispersion rather than chemical bonding. However, at higher loading (3 wt.%), scanning electron microscopy with energy-dispersive X-ray spectroscopy (SEM-EDX) revealed notable nanoparticle aggregation. Functionally, the multilayer films demonstrated markedly improved water vapor barrier properties compared to single-layer PBAT/TPS films. Migration studies showed that silver release increased with nanoparticle concentration and was significantly enhanced under acidic conditions relative to distilled water. Importantly, Ag-NP-incorporated laminates exhibited pronounced antibacterial activity against *Staphylococcus aureus*. Collectively, these findings highlight the potential of Ag-NP-enriched, starch-based multilayer laminates as next-generation active packaging systems that combine with effective microbial control.

## 1. Introduction

Active packaging systems are designed to preserve product quality, enhance safety, and extend shelf life through the use of materials or technologies with specific functional properties. Such systems contribute to reductions in food waste and environmental impact, thereby supporting improved sustainability [1]. However, the relatively high cost of single-use biodegradable materials continues to limit their broader commercial application across various products. To address this limitation, blends based on thermoplastic starch (TPS), a cost-effective biodegradable polymer, have been developed. TPS-based blends have been widely investigated, including PBAT/TPS [2,3], PLA/TPS [4,5], and PBS/TPS systems [6,7]. The incorporation of functional additives has been reported to effectively modify the microstructure, morphology, and overall performance of these materials. Despite these advances, the barrier properties of biodegradable polymers generally remain insufficient when compared to conventional plastics. Therefore, systematic studies on multilayer film structures are necessary to better understand how additive incorporation influences morphology and performance, and to support the development of more effective bioplastic-based active packaging systems. A multilayer laminate structure was designed to combine the complementary properties of different biodegradable polymers. Differences in polymer structure and diffusion characteristics between layers enhance resistance to oxygen and moisture permeation [8,9]. The PLA layer supplies mechanical rigidity and barrier protection, while the inner PBAT/TPS layer serves as the active interface. Previous studies have not reported the fabrication of multilayer packaging composed entirely of biodegradable materials using an extrusion-based process.

Pure silver is known for its high thermal and electrical conductivity, as well as its relatively low contact resistance. Silver nanoparticles (Ag-NPs) have been widely used as antimicrobial agents due to their ability to inhibit bacterial growth. Owing to their nanoscale size, Ag-NPs possess a large surface area, resulting in high surface energy and an increased number of reactive sites. The antimicrobial activity of silver nanoparticles is primarily attributed to the release of Ag^+^ ions, which can disrupt bacterial cell membranes and induce the formation of reactive oxygen species. These processes interfere with essential cellular functions, including DNA replication and protein synthesis, ultimately leading to bacterial inactivation [10,11]. Numerous studies have demonstrated that polymeric constituents, including PBAT and PLA, are widely incorporated as filler [12,13].

Food is susceptible to microbial spoilage during storage. The extent and rate of spoilage depend on several factors, including the type of product, initial microbial load, oxygen exposure, moisture content, and storage temperature. Microbial proliferation and lipid oxidation are the primary mechanisms responsible for deterioration, leading to off-odors, discoloration, and textural changes [14,15]. Conventional packaging technologies, such as vacuum packaging, modified atmosphere packaging (MAP), and the use of oxygen scavengers, are commonly applied to maintain food quality by controlling oxygen availability, regulating moisture, and limiting microbial growth. More recently, increasing attention has been directed toward active packaging systems incorporating antimicrobial agents, which can directly inhibit pathogenic and spoilage microorganisms and thereby extend the shelf life of food products [16,17].

This study contributes to ongoing efforts to enhance the functional performance of laminated plastics for active food packaging applications. The key innovation is that the PLA outer layer provides mechanical rigidity and moisture resistance, and the PBAT blend TPS with an Ag-NP inner layer serves as the active antimicrobial interface. It is hypothesized that incorporating Ag-NPs into bioplastic-based packaging systems can effectively inhibit bacterial growth in food products. This antimicrobial effect is expected to result from the combined contribution of the multilayer structure, which provides improved barrier properties, and the controlled release of antimicrobial agents upon direct contact. By integrating these functionalities, this work seeks to develop novel active packaging materials with enhanced performance, improved sustainability, and practical com-mercial potential through extrusion and lamination processes. In addition, the study explores the potential synergistic interactions between antimicrobial agents and bio-degradable polymer matrices, aiming to enable more precise tailoring of material properties to meet the evolving demands of the active packaging industry.

## 2. Materials and Methods

### 2.1. Laminate Sheets Preparation

#### 2.1.1. Ag-NPs Films Preparation

**Figure 1:**
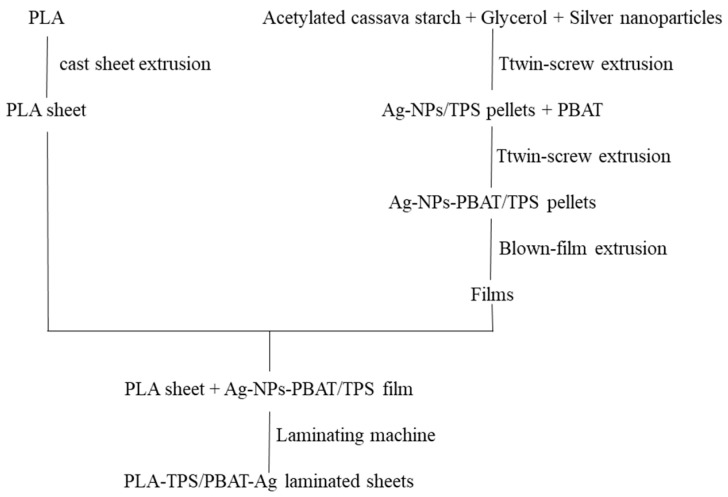
Scheme of the steps of fabrication process of the laminated sheets.

Silver nanoparticles (Prime Nanotechnology Co., Ltd., Bangkok, Thailand) were incorporated into thermoplastic starch (TPS)/PBAT matrices (Figure 1) to prepare Ag-NP-loaded composite films. The TPS phase was initially prepared by compounding acetylated cassava starch (AS; degree of substitution 0.01–0.03, Siam Modified Starch Co., Ltd., Pathum Thani, Thailand) with glycerol (Asian Scientific Co., Ltd., Samut Prakan, Thailand) at a solid-to-glycerol weight ratio of 100:35. The starch and glycerol were premixed using a dough mixer for approximately 10 min to promote uniform distribution prior to extrusion. The blended mixture was then fed into a twin-screw extruder (LVF2202–130, L/D ratio = 48, screw diameter = 20 mm; Labtech Engineering Co., Ltd., Samut Prakan, Thailand) within 60 min after mixing. Extrusion was carried out under a controlled temperature profile of 95/100/115/120/125/130/135/135/140/140/145/150 °C at a screw speed of 200 rpm to obtain the TPS phase.

Composite Ag-NP–PBAT/TPS pellets were prepared by blending PBAT (Ecoflex^®^ F Blend C1200, BASF, Ludwigshafen, Germany) with the previously prepared TPS and silver nanoparticles at loadings of 0, 1, 2, and 3 wt.%, while maintaining a TPS:PBAT weight ratio of 60:40. The blending process was carried out using a twin-screw extruder under a temperature profile of 95/110/115/120/125/130/135/140/145/145/150/155 °C at a screw speed of 180 rpm. The resulting composite pellets were subsequently processed into films using a single-screw blown-film extruder (LF-400, L/D ratio = 30, screw diameter = 25 mm; Labtech Engineering Co., Ltd., Thailand). The barrel temperature was set between 145 and 160 °C. The screw speed and take-up roller speed were maintained at 45 rpm and 3 m/min, respectively. Airflow was carefully controlled to ensure the formation of a stable and uniformly inflated bubble. The obtained films were stored at ambient conditions in Ziploc bags prior to further characterization.

#### 2.1.2. PLA Sheets Preparation

Poly(lactic acid) (PLA; NatureWorks^®^ Ingeo 2003D, extrusion grade; NatureWorks LLC, Plymouth, MN, USA) sheets were fabricated using a single-screw extruder (Labtech Engineering Co., Ltd., Samut Prakan, Thailand) under controlled processing conditions. The extrusion temperature profile was set at 160/160/170/175/180/185/185 °C, and the screw speed was maintained at 50 rpm. The cast-sheet extrusion process produced uniform sheets with a thickness ranging from 0.30 to 0.35 mm. The prepared PLA sheets were stored in sealed aluminum-laminated bags at ambient temperature prior to lamination and subsequent characterization.

#### 2.1.3. Laminate Sheets Preparation

Samples of TPS/PBAT- Ag-NP film and PLA sheet were cut to dimensions of 22 cm × 25 cm and assembled by stacking the layers together on a backing sheet. The assembled films were first passed through rollers and subsequently laminated using a laminating machine (Laminator vision 330C, Hangzhou Befitter Machinery & Electronic Co., Ltd., Hangzhou, China) at 95 °C in both forward and reverse directions. After lamination, the PLA–TPS/PBAT–Ag-NP sheets were allowed to cool at room temperature until fully stabilized. The laminated sheets were then sealed and stored in aluminum-laminated bags at room temperature prior to further use.

### 2.2. Characterization of the Active Sheets

#### 2.2.1. Scanning Electron Microscopy (SEM)

The microstructure of the films was examined using a scanning electron microscope (SEM; SU3500, Hitachi, Tokyo, Japan) operated at an accelerating voltage of 10 kV. Prior to observation, the films were fractured in liquid nitrogen and subsequently coated with a thin layer of gold using a sputter coater (MSP-1S, Vacuum Device Inc., Mito, Japan).

#### 2.2.2. Light Transmission

The optical transmittance of the samples (3 × 4 cm) was measured using a UV–visible spectrophotometer (Evolution 300, Thermo Fisher Scientific, Madison, WI, USA) over a wavelength range of 200–800 nm at a scanning rate of 600 nm·min^−1^. All measurements were conducted in triplicate, and the results are presented as the mean of three independent determinations.

#### 2.2.3. Fourier-Transform Infrared Spectroscopy (FTIR)

The infrared absorption spectra of the films were obtained using attenuated total reflectance Fourier-transform infrared spectroscopy (ATR-FTIR; Tensor 27, Bruker Optics GmbH, Ettlingen, Germany). The spectra were recorded over a wavenumber range of 500–4000 cm^−1^ at a resolution of 4 cm^−1^. For each sample, three replicates were collected and averaged over 64 scans.

#### 2.2.4. Water Vapor Permeability

The water vapor transmission rate (WVTR) of the films was determined using the standard cup method (ASTM E96/E96M-12). Anhydrous silica gel was placed as a desiccant in metal cups, which were covered with circular film specimens (7 cm in diameter). The edges of the specimens were sealed with paraffin wax and O-rings to ensure airtight conditions. The assembled cups were stored in a controlled-humidity chamber (Binder KBF 720, GmbH, Ettlingen, Germany) at 25 ± 2 °C and 50 ± 2% relative humidity. The cups were weighed at regular intervals until a steady-state weight gain was achieved. The WVTR (g·m^−2^·day^−1^) was calculated from the slope of the linear portion of the weight gain versus time curve. Water vapor permeability (WVP; g·mm·Pa^−1^·m^−2^·day^−1^) was subsequently determined using Equation (1).

(1) $$\mathrm{WVP}=\frac{\mathrm{WVTR}\times L}{\Delta P}$$

where *L* is the film thickness (mm), and ΔP represents the water vapor pressure difference (Pa) across the film. Each reported value represents the mean of five independent measurements.

#### 2.2.5. Oxygen Permeability

The oxygen transmission rate (OTR) of the films was measured using an oxygen permeation analyzer (Model 2/22H, Mocon, Minneapolis, MN, USA) in accordance with ASTM D3985. Prior to testing, circular film specimens (14 cm in diameter) were conditioned for 48 h at 25 °C and 50% relative humidity. The reported values represent the mean of four independent measurements. Oxygen permeability (OP) was calculated using Equation (2) and expressed in units of cm^3^·mm·m^−2^·day^−1^·kPa^−1^.

(2) $$\mathrm{OP}=\frac{\mathrm{OTR}\times L}{\Delta P}$$

where *L* denotes the film thickness (mm) and *ΔP* represents the difference in oxygen partial pressure across the film or sheet.

#### 2.2.6. Mechanical Properties

Tensile strength (TS), elongation at break (EB), and Young’s modulus (YM) were determined using an Instron Universal Testing Machine (Model 5965, Instron, Norwood, MA, USA) in accordance with ASTM D882-10 (2000). Prior to testing, film specimens (150 × 25 mm) were conditioned at 50% relative humidity for 48 h. The reported values represent the mean of ten replicate measurements.

#### 2.2.7. Specific Ag-NP Migration

In accordance with Commission Regulation (EU) No. 10/2011, sheets containing Ag-NPs were cut into specimens (5 × 5 cm) and subjected to migration testing using appropriate food simulants. The samples were immersed in 3% (*v*/*v*) acetic acid and in distilled water (120 mL and 100 mL, respectively). All specimens were maintained in contact with the simulants at 5 ± 2 °C for 10 days to simulate refrigerated storage conditions. After the exposure period, the sheets were removed and the simulant solutions were collected for analysis. The specific migration of silver was quantified using inductively coupled plasma–optical emission spectrometry (ICP-OES; Agilent 5100 SVDV ICP-OES, Agilent Technologies Inc., Santa Clara, CA, USA). The operating conditions were as follows: nebulizer flow rate, 0.8 L·min^−1^; plasma gas flow rate, 12 L·min^−1^; auxiliary gas flow rate, 1 L·min^−1^; viewing height, 8 mm; RF power, 1.20 kW; read time, 5 s; and stabilization time, 20 s. Silver migration was expressed as mg Ag·dm^−2^ of film surface area.

#### 2.2.8. Antibacterial Activity

Antibacterial activity of the laminate sheets was evaluated by monitoring changes in optical density (OD). *S. aureus* was cultured in nutrient broth (NB) at 37 °C for 24 h to prepare the bacterial inoculum. The bacterial suspension was then diluted by mixing 1 mL of the culture with 9 mL of sterile 0.1% peptone solution. Approximately 1 g of sheet specimens was immersed in the inoculated peptone medium, corresponding to an initial bacterial load of approximately 7 log CFU·g^−1^ for *S. aureus*. The samples were incubated at 37 °C, and aliquots of the supernatant were collected at 0, 1, 2, and 3 days for OD measurement. Absorbance at 620 nm was determined using a UV–visible spectrophotometer (Spectronic Genesys 10 UV, Thermo Fisher Scientific, Waltham, MA, USA). The change in optical density (ΔOD_620_) at each sampling time was calculated as the difference between the absorbance after incubation (t_n_) and the initial absorbance (t_0_). In addition, after 24 h of incubation, aliquots were plated on nutrient agar to determine viable cell counts (CFU·mL^−1^) and to confirm the antibacterial activity of the laminate sheets.

#### 2.2.9. Statistical Analysis

All experimental data were analyzed using IBM SPSS Statistics software (version 22; IBM Corp., New York, NY, USA). The effect of silver content on the measured parameters was evaluated by one-way analysis of variance (ANOVA). When significant differences were detected, mean comparisons were performed using Duncan’s multiple range test. Statistical significance was established at a 95% confidence level (*p* < 0.05).

## 3. Results

### 3.1. Appearance of Laminate Sheets

#### 3.1.1. Microstructures

**Figure 2:**
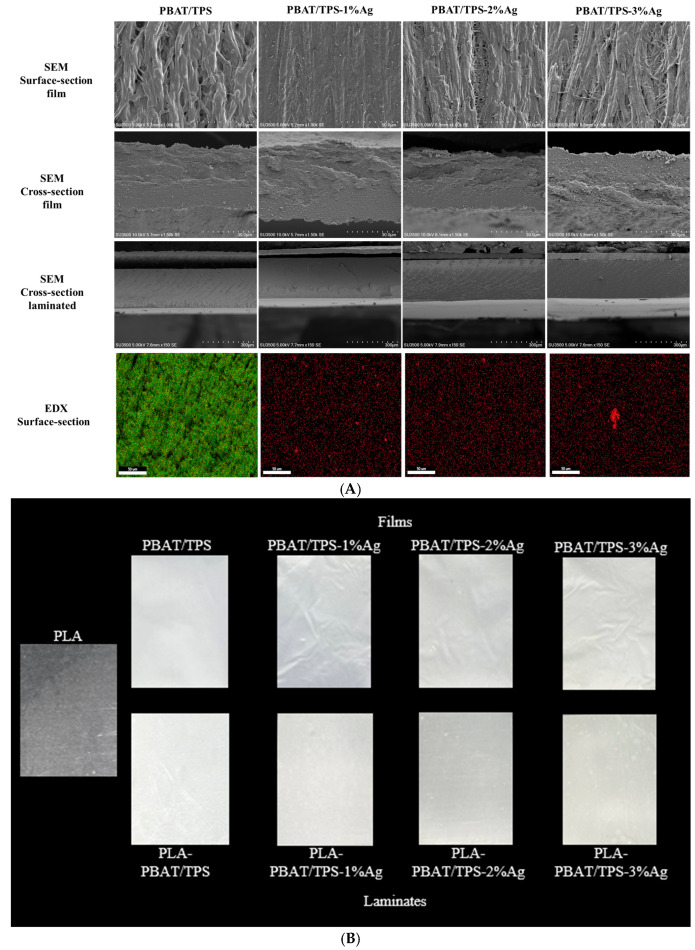

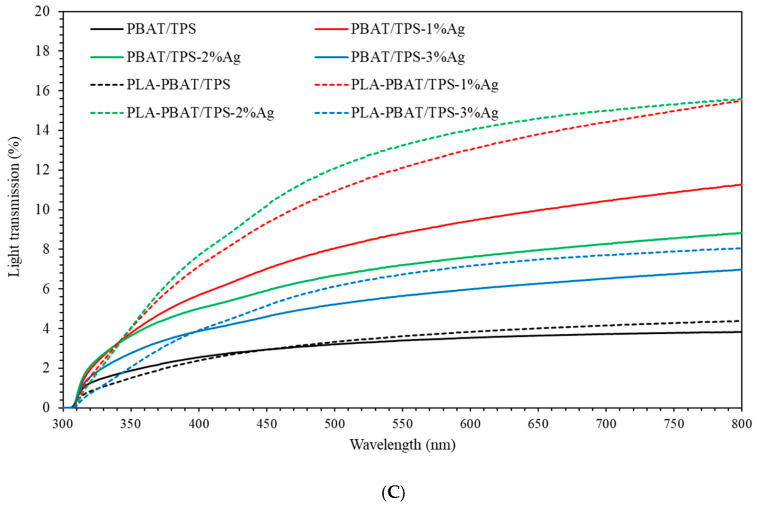
(**A**) Microstructures, (**B**) appearance, and (**C**) light transmission of PLA-PBAT/TPS laminates sheets containing silver at 0, 1, 2 and 3 wt.%.

The SEM micrographs shown in Figure 2A reveal distinct differences in surface morphology and cross-sectional structure among the composite laminates. The unlaminated substrate exhibited a fiber-like structure, which is characteristic of hydrophilic and mechanically heterogeneous film layers. This morphology suggests limited miscibility between the PBAT and TPS phases, leading to phase separation [18]. The oriented fibrous domains appear to follow the flow direction generated during the blown-film extrusion process. Due to the polarity difference between TPS (polar) and PBAT (non-polar), blending these polymers results in partial phase separation [19]. The observed surface densification is consistent with previous reports on TPS/PBAT blown films, where hydrogen bonding interactions and physical entanglement contribute to improved structural cohesion at the surface [20]. The incorporation of 1 wt.% Ag-NPs improved the uniform dispersion of the fibrous TPS domains within the PBAT matrix, indicating enhanced interfacial compatibility. At 3 wt.% Ag-NPs, the surface morphology became more compact, with increased apparent fiber density, which may be attributed to nanoparticle aggregation and altered phase organization. These morphological changes suggest that Ag-NP loading influences phase distribution and surface structural integrity.

Cross-sectional SEM images showed relatively smooth fracture surfaces across all samples, consistent with the uniform appearance observed on the top surface. Small pores were distributed throughout the matrix, with a slight increase in pore size as the Ag-NP content increased. However, the sample containing 3 wt.% Ag-NPs did not exhibit any pronounced structural irregularities. The enlargement of pores at higher Ag-NP loadings may be associated with increased moisture retention during film formation, followed by evaporation during extrusion and subsequent solidification. The crust-like surface features observed are therefore likely related to water loss and localized structural collapse during processing [21].

#### 3.1.2. Energy Dispersive X-Ray Spectroscopy (EDX)

The distribution of silver within the laminate structure is clearly observed in the EDX elemental mapping (Figure 2A, bottom row). In the control sample, only a uniform background signal corresponding to the polymer matrix (green channel) is detected. In contrast, the silver-containing films exhibit a distinct red signal distributed throughout the matrix, indicating the presence of Ag-NPs. The mapping results confirm that silver was successfully incorporated into the polymer network, although variations in signal intensity were observed among different formulations. The film containing 3 wt.% Ag-NPs shows localized areas of particle aggregation, which become more apparent at higher loadings. Nevertheless, the predominantly dispersed and finely distributed red signals suggest that silver is embedded within the matrix at the micro- to submicrometer scale.

#### 3.1.3. Light Transmissions

The optical properties of TPS/PBAT-Ag-NP blend films and PBAT/TPS-Ag-NP blends laminated with PLA films at concentrations ranging from 0 to 3 wt.% are presented in Figure 2B,C. All samples exhibited a certain degree of transparency. The incorporation of Ag-NPs influenced the light transmittance of the films, with noticeable changes observed as the Ag-NP content increased. At higher Ag-NP concentrations, light scattering effects became more pronounced, which may be attributed to nanoparticle-induced scattering within the polymer matrix [22]. These observations are consistent with the EDX results, which indicated differences in Ag-NP distribution among the formulations. Similarly, in the laminated PBAT/TPS blends laminated with PLA films, increasing Ag-NP content (1, 2, and 3 wt.%) resulted in progressively higher light transmittance, with values approximately 2-, 6-, and 7-fold greater than those of the control, respectively.

### 3.2. Fourier-Transform Infrared Spectroscopy

**Figure 3:**
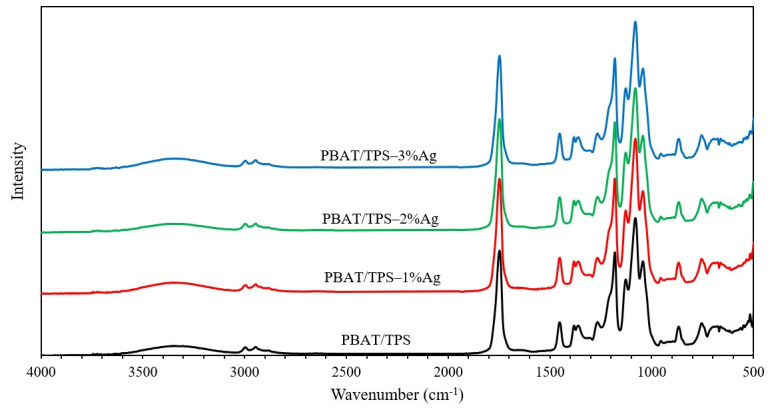
FTIR absorption spectra of PBAT/TPS films containing silver at 0, 1, 2 and 3 wt.%.

Fourier-transform infrared (FTIR) analysis revealed no significant chemical interactions between Ag-NPs and the starch/PBAT matrix. The FTIR spectra of PBAT/TPS blends films containing 0–3 wt.% Ag-NPs are presented in Figure 3. Characteristic absorption bands of PBAT were observed, including C–H stretching vibrations in the range of 2950–2870 cm^−1^, ester carbonyl (C=O) stretching at approximately 1710–1722 cm^−1^, aromatic C=C stretching at 1600–1505 cm^−1^, and C–O–C stretching within the 1250–1100 cm^−1^ region [23]. No new absorption peaks or noticeable shifts in peak position were detected upon Ag-NP incorporation, indicating that the addition of Ag-NPs did not alter the fundamental chemical structure of the polymer matrix. The broad absorption band observed between 3000 and 3700 cm^−1^ in the starch control film is attributed to the O–H stretching vibration characteristic of the TPS phase. Intra- and intermolecular hydrogen bonding, which is dependent on the bond energies and the distance between hydrogen and oxygen atoms, is responsible for the location of the O-H stretching peak [6,24].

### 3.3. Water Vapor Permeability (WVP)

**Figure 4:**
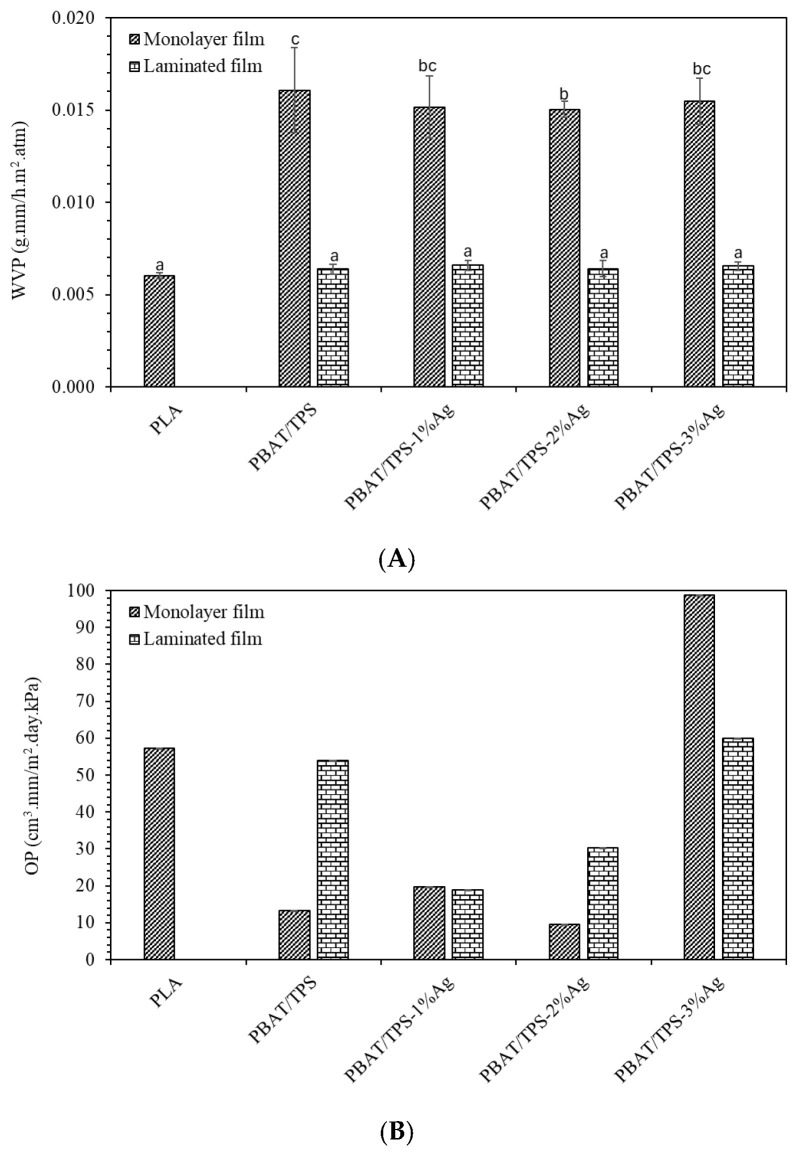
(**A**) Water permeability (WVP) and (**B**) oxygen permeability (OP) of PLA-PBAT/TPS laminates containing silver at 0, 1, 2 and 3 wt.%.

Figure 4A presents the water vapor permeability (WVP) values of TPS/PBAT blends and PBAT/TPS blends laminated with PLA films containing 0–3 wt.% Ag-NPs. The control film (TPS/PBAT without Ag-NPs) exhibited the highest WVP, which can be attributed to the hydrophilic nature of starch. The abundance of hydroxyl groups in starch promotes water vapor sorption and facilitates diffusion through the polymer matrix [20]. Increasing the Ag-NP content led to a reduction in WVP [8,9,25], likely due to the formation of a denser microstructure and the barrier effect created by dispersed silver nanoparticles, which hindered water vapor diffusion. In contrast, upon lamination with PLA, the composite films displayed WVP values comparable to those of neat PLA across all Ag-NP concentrations. This behavior is associated with the dense molecular structure and relatively hydrophobic nature of PLA, which effectively limits water vapor penetration [26].

### 3.4. Oxygen Permeability (OP)

Figure 4B presents the oxygen permeability (OP) values of TPS/PBAT blends and PBAT/TPS blends laminated with PLA films containing Ag-NPs at concentrations up to 3 wt.%. An increase in Ag content was associated with higher OP values, particularly at 3 wt.%. This trend may be attributed to nanoparticle aggregation at higher loadings, which can introduce microstructural defects or discontinuities within the polymer matrix, thereby facilitating oxygen transport. The 3 wt.% Ag-NPs level appears to exceed the optimal concentration for maintaining barrier integrity. In contrast, when laminated with PLA, the composite films exhibited OP values comparable to those of neat PLA across all Ag-NP concentrations. This behavior is attributed to the dense molecular structure and relatively low oxygen permeability of PLA, which effectively restricts gas diffusion [26].

### 3.5. Mechanical Properties

#### 3.5.1. Monolayer Films

**Figure 5:**
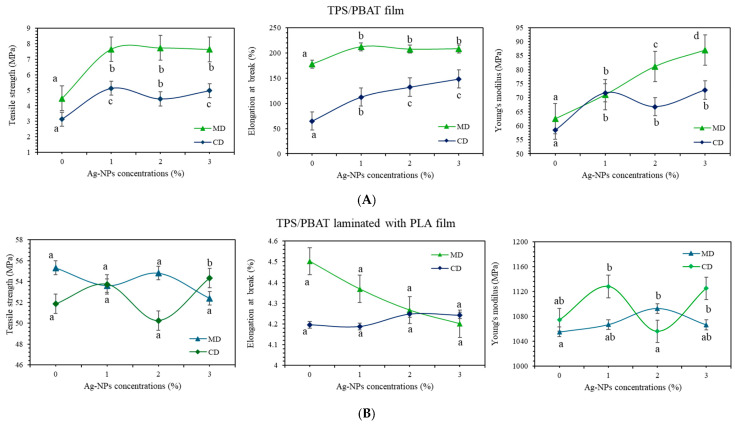
Tensile properties as tensile strength (TS) elongation at break (EB) and Young’s modulus (YM) of (**A**) PBAT/TPS films containing silver at 0, 1, 2 and 3 wt.% and (**B**) PLA-PBAT/TPS laminates containing silver at 0, 1, 2 and 3 wt.%.

The mechanical properties of the blown films, including tensile strength (TS), elongation at break (EB), and Young’s modulus (YM), measured in both the machine direction (MD) and cross direction (CD), are presented in Figure 5A. The films exhibited higher mechanical performance in the MD compared to the CD, which is commonly attributed to molecular orientation induced during the blown-film extrusion process. Both MD and CD values of TS, EB, and YM showed an increasing trend with Ag-NP incorporation, suggesting a reinforcing effect of the silver nanoparticles. Well-dispersed Ag-NPs may enhance stress transfer within the polymer matrix and improve structural integrity. In addition, appropriate nanoparticle dispersion can contribute to improved chain interaction and mobility, thereby influencing flexibility and mechanical behavior [27,28].

#### 3.5.2. Laminated Sheets

Figure 5B presents the mechanical properties of the PBAT/TPS blends laminated with PLA films containing Ag-NPs, including tensile strength (TS), elongation at break (EB), and Young’s modulus (YM). The laminated structures exhibited enhanced TS, EB, and YM values in both the machine direction (MD) and cross direction (CD). The improved tensile performance is primarily attributed to the contribution of the PLA outer layer, which provides structural rigidity and load-bearing capacity [9,12,29]. The observed differences between MD and CD reflect the anisotropic nature of the laminated structure, arising from the heterogeneous combination of two distinct polymer layers [30].

### 3.6. Impact Strength and Adhesive Peel Strength

#### 3.6.1. Impact Strength

**Figure 6:**
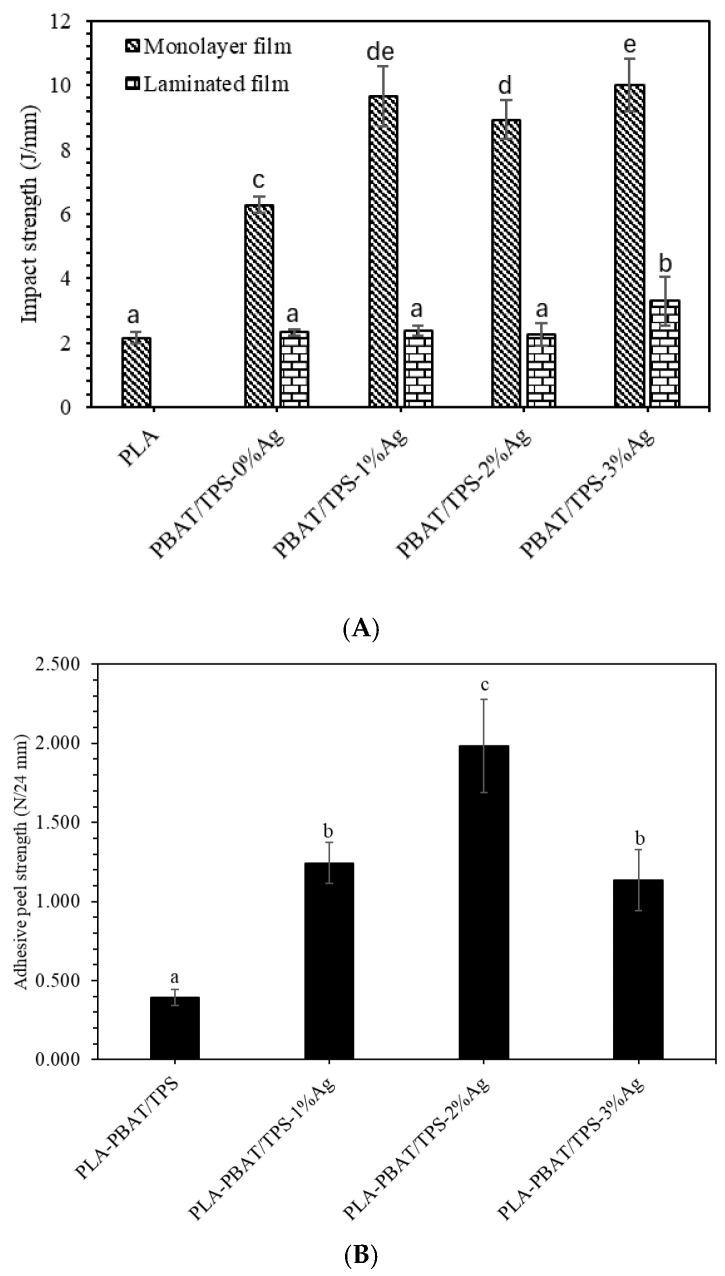
(**A**) Impact strength of PBAT/TPS films containing silver at 0, 1, 2 and 3 wt.% and PLA-PBAT/TPS sheets containing silver at 0, 1, 2 and 3 wt.% and (**B**) adhesive peel strength of PLA-PBAT/TPS laminates containing silver at 0, 1, 2 and 3 wt.%.

Figure 6A presents the impact strength of TPS/PBAT blends and PBAT/TPS blends laminated with PLA films containing silver at concentrations ranging from 0 to 3 wt.%. The results indicate that the PBAT/TPS films exhibited higher impact resistance than the PLA–PBAT/TPS laminates. In particular, the PBAT/TPS film containing 3 wt.% Ag-NPs showed the highest impact strength, which may be attributed to the inherent viscoelastic behavior of PBAT that allows effective energy absorption during impact [31]. In addition, the incorporation of Ag-NPs may contribute to improved energy dissipation within the polymer matrix by acting as stress distribution sites [32]. In contrast, the laminated PLA–PBAT/TPS structures demonstrated lower impact resistance. This reduction is likely associated with the relatively brittle nature of the PLA layer, which dominates the mechanical response of the laminate and limits its ability to absorb impact energy [33].

#### 3.6.2. Adhesive Peel Strength

Figure 6B presents the adhesive peel strength of PBAT/TPS blends laminated with PLA films containing Ag-NPs at concentrations ranging from 0 to 3 wt.%. The laminate without Ag-NPs (0 wt.%) exhibited the lowest peel strength. This result suggests limited interfacial compatibility between the PLA and PBAT/TPS layers. The difference in polarity between hydrophobic PLA and the more hydrophilic TPS component likely contributes to weaker interfacial adhesion [33]. When Ag-NPs were incorporated at 3 wt.%, a reduction in interfacial shear strength between the laminate layers was observed. This decrease is likely associated with nanoparticle aggregation at higher loadings, suggesting that 3 wt.% exceeds the optimal concentration for maintaining effective interfacial adhesion [34,35].

### 3.7. Specific Migration of Silver Analyzed

**Figure 7:**
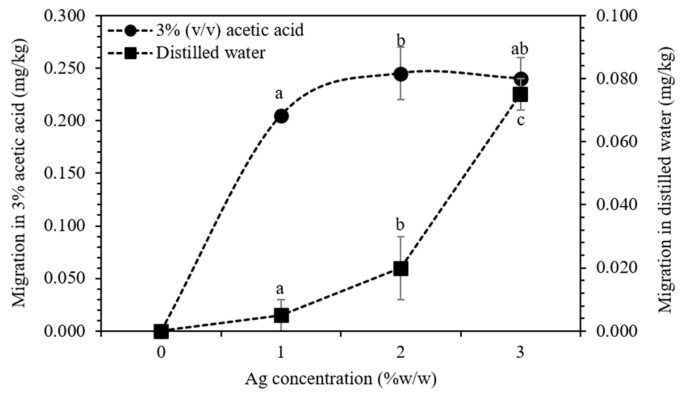
Specific migration of silver metal from PLA-PBAT/TPS sheets containing silver at 0, 1, 2 and 3 wt.% into food simulants water and 3% (*v*/*v*) acetic acid. Different letters indicate significant differences among sheets containing different Ag-NP concentrations (*p* ≤ 0.05).

The specific migration of silver from PBAT/TPS blends laminated with PLA films containing 0–3 wt.% Ag-NPs in distilled water and 3% acetic acid food simulants is presented in Figure 7. The results indicate that silver migration increased with increasing Ag-NP content in the films. Migration levels were higher in 3% acetic acid compared to distilled water. This behavior can be attributed to the enhanced release of Ag^+^ ions under acidic conditions, as well as the swelling of the TPS phase, which may facilitate diffusion of silver species from the polymer matrix [35,36].

After conversion to mg/kg using the standard surface area-to-volume ratio (6 dm^2^/kg), the migration values of the 1 and 2 wt.% Ag-NP formulations under distilled water remained below the SML of 0.05 mg/kg. The 3 wt.% formulation exceeded the regulatory limit under distilled water conditions, whereas the 1–3 wt.% formulation exceeded the SML in 3% (*v*/*v*) acetic acid simulant. These findings indicate that silver loading should be carefully optimized to ensure compliance, particularly for applications involving acidic foods.

### 3.8. Antibacterial Activities

**Figure 8:**
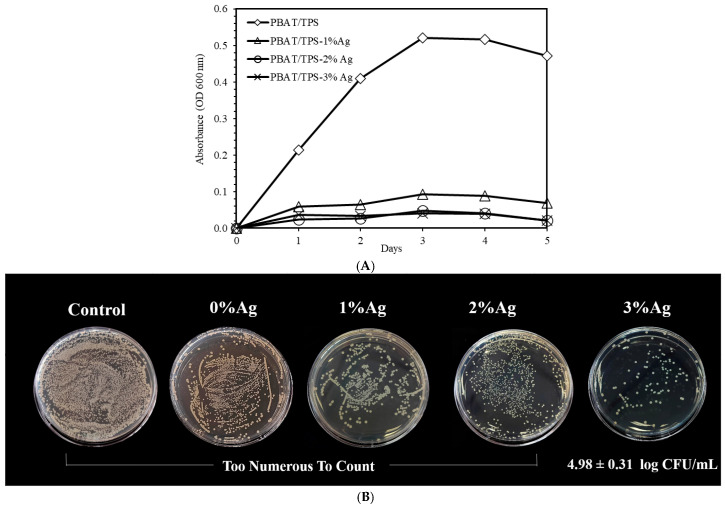
(**A**) Optical density for *Staphylococcus aureus* during incubation for 5 days; (**B**) viable plate count of *Staphylococcus aureus* during incubation for 24 h of PLA-PBAT/TPS laminates containing silver at 0, 1, 2 and 3 wt.%.

The antibacterial activity of PBAT/TPS blends films containing Ag-NPs at concentrations ranging from 0 to 3 wt.% against *S. aureus* in liquid medium is presented in Figure 8A. During the five-day incubation period, the control PBAT/TPS films exhibited a continuous increase in absorbance, indicating rapid bacterial growth. In contrast, films containing Ag-NPs showed lower absorbance values, reflecting a reduction in microbial proliferation over time. The extent of growth inhibition increased with higher Ag-NP content. This effect is attributed to the release of Ag^+^ ions, which can disrupt bacterial cell membranes, denature intracellular proteins, and interfere with DNA replication, ultimately inhibiting bacterial growth [37,38].

Figure 8B presents the number of bacterial colonies in the culture medium after immersion of PBAT/TPS films containing 0–3 wt.% Ag-NPs for 24 h. In the medium inoculated with *S. aureus*, the film containing 3 wt.% Ag-NPs exhibited the strongest antibacterial effect, as evidenced by the lowest colony count (4.98 ± 0.31 log CFU/mL). The antimicrobial activity is attributed to the release of Ag^+^ ions from the film into the surrounding medium. These ions interact with bacterial cell membranes, increasing membrane permeability and disrupting cellular integrity, ultimately leading to bacterial inactivation [37,38,39].

## 4. Conclusions

This study presents the successful development of an active, multilayer packaging system by laminating a PLA outer layer with a PBAT/TPS inner film functionalized with Ag-NPs. Incorporation of silver nanoparticles at concentrations up to 2 wt.% significantly enhanced mechanical strength, interfacial compatibility, and antibacterial efficacy against *Staphylococcus aureus*, while maintaining the structural integrity of the polymer matrix, as evidenced by FTIR analysis. The PLA outer layer played a critical role in reinforcing barrier performance, markedly reducing water vapor compared to monolayer PBAT/TPS systems. Migration studies further revealed that silver release is governed by both nanoparticle concentration and the surrounding medium, with elevated migration observed under acidic conditions. At higher loading (3 wt.% Ag-NPs), nanoparticle aggregation became evident, leading to performance deterioration beyond the optimal dispersion threshold and potentially compromising structural uniformity, barrier integrity, interfacial performance, and regulatory compliance. In contrast, the 1 wt.% Ag-NPs provided a balanced performance profile, demonstrating significant antibacterial activity, improved mechanical strength and peel resistance, enhanced moisture barrier properties, and better nanoparticle dispersion. This formulation represents the best compromise between functional performance and safety considerations.

## Data Availability

Data presented in this study are available on reasonable request from the corresponding author.
